# A rare case of autoimmune limbic encephalitis: an uncharted territory!

**DOI:** 10.17712/nsj.2017.4.20170150

**Published:** 2017-10

**Authors:** Hatim Ibrahim, Abdulelah N. Al Jasser, Sonia A. Khan, Kalthoum G. Tlili

**Affiliations:** *From the Department of Neurology (Khan, Ibrahim), Department of Radiology (Tlili), Prince Sultan Military Medical City, and from the Faculty of Medicine (Aljasser), King Saud University, Riyadh, Kingdom of Saudi Arabia*

## Abstract

Autoimmune encephalitis is rare. Several auto- antibodies are described in autoimmune encephalitis. We describe a case of autoimmune limbic encephalitis associated with positive voltage gated potassium channel (VGKC) antibodies and positive leucine-rich glioma inactivated protein 1 antibodies (LGI1). A 33-year-old Saudi housewife, she presented with 2 months history of cognitive deterioration and recurrent left facio-brachial dystonic seizures followed by generalized tonic clonic seizures. At times the seizures are preceded by rising epigastric aura and shortness of breath. The neurological examination was normal apart from upgoing left plantar reflex. She had borderline IQ of 76 with impaired verbal fluency and impaired visual and verbal memory. Magnetic resonance imaging of the brain showed right mesial temporal non-enhancing lesion. Cerebrospinal fluid examination was positive for LGI1 and VGKC. Optimal seizure control was achieved with immunotherapy.

Autoimmune limbic encephalitis has been described as a paraneoplastic syndrome associated with anti neural antibodies produced by tumors against intracellular antigens.^1^ The classical clinical presentation includes subacute cognitive deterioration, seizures and psychosis.^1^ Recently, autoimmune encephalitis not related to tumors and with antibodies targeting extracellular antigens is described in several case reports and named the neuronal surface antibody syndrome or autoimmune synaptic encephalitis.^2^ Those neuronal surface auto antibodies are directed against the neuronal cell surface or the synaptic proteins namely N-methyl-D-aspartate, α-amino-3-hydroxy-5-methyl-isoxazoleproionic acid, and γ-aminobutyric acid B receptors.^2^ Leucine-rich glioma-inactivated protein 1 (LGI1) autoantibodies are increasingly described in cases of autoimmune encephalitis not related to tumors and thought previously to be related to antibodies against the voltage gated potassium channel (VGKC).^2^ In this report, we describe a case of autoimmune limbic encephalitis associated with positive voltage gated potassium channel antibodies VGKC and positive leucine-rich glioma inactivated protein 1 antibodies LGI1.

## Case Report

**Figure 1:**
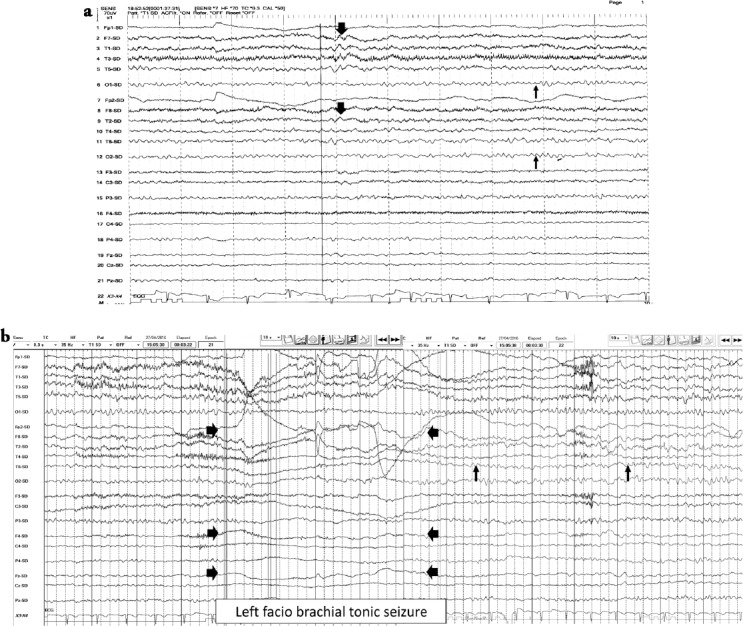
EEG features a) Interictal EEG showed normal EEG background of 8-9 hertz bilaterally (thin arrows). Intermixed slow transients of 6-7 hertz in the temporal regions bilaterally (thick arrows). No epileptiform discharges seen, b) The ictal EEG showed periods of right hemispheric electro-decremental response with right hemispheric alpha frequency attenuation at FP2, F4, T2, T4, T6, P4 & O2 with some diffusion to the left for 4 seconds time locked with left facio brachial tonic seizure (thick arrows) followed by recovery of the EEG background intermixed with right temporal slow transients of 4-5 hertz at T2, T4 and T6 (thin arrows).

**Figure 2:**
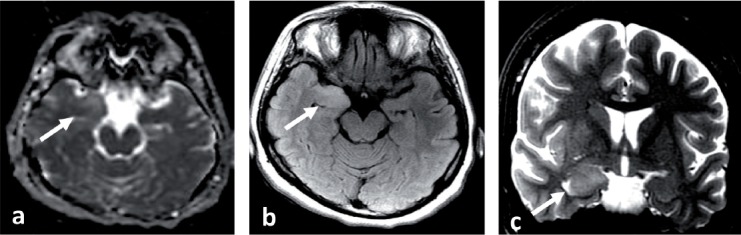
MRI brain at presentation **a)** Axial apparent diffusion coefficient (ADC) map, **b)** Axial fluid attenuated inversion recovery (FLAIR) and **c)** Coronal T2 spin echo (T2SE) images showing abnormal high T2 and FLAIR signal intensity and swelling with blurring of the margins of right amygdala and medial right temporal lobe cortex and increased diffusivity associated on ADC map (White arrows). No abnormal enhancement (post contrast images not shown).

**Figure 3:**
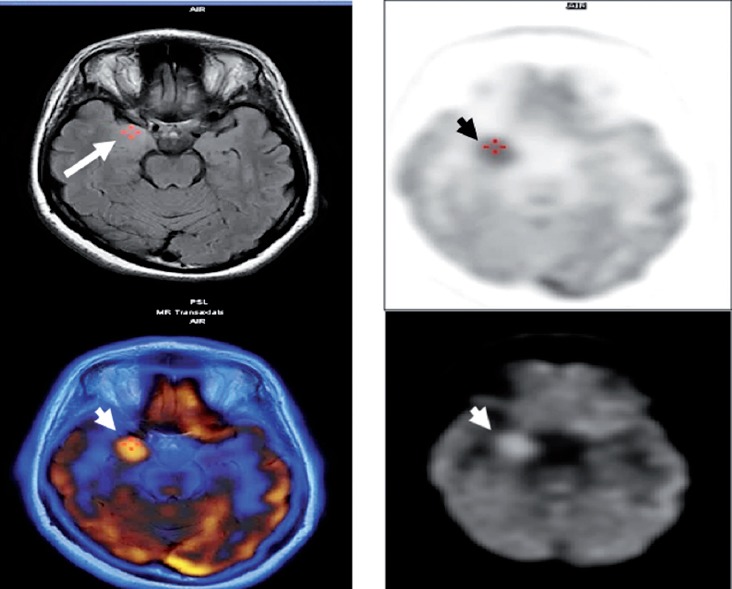
PET scan of the brain at presentation showed hypermetabolic right mesial temporal area (White & black arrow heads) which matched with MRI abnormality (White arrow).

A 33-year-old Saudi housewife from the north of Saudi Arabia, right handed with no epilepsy risk factors and no chronic medical illnesses. She presented with history of recurrent attacks of left facio-brachial dystonic seizures, which progressed in 2 months to generalized tonic clonic seizures. At times the focal motor seizures are preceded by a rising abdominal aura or shortness of breath. She also had behavioral and cognitive deterioration and was noticed by her family to be less interactive with poor memory for 2 months. The neurological examination revealed no motor or sensory deficit except for left up going plantar reflex. Neuropsychological evaluation revealed a borderline IQ of 76 with impaired verbal fluency and impaired visual and verbal memory. The rest of the physical examination was normal. Blood investigations including renal function, sodium and other electrolytes, liver profile, thyroid function test, thyroid antibodies, tumor markers, paraneoplastic autoantibodies and vasculitis screen where within normal range. Cerebro-spinal fluid (CSF) analysis showed 2 WBC with mild elevation of the protein of 0.55 g/L (Normal 0.35-0.45 g/L), normal glucose and negative staining and cultures for bacteria and acid fast bacilli. Herpes simplex polymerase chain reaction was negative. Leucine-rich glioma-inactivated protein 1 auto antibodies titer in the serum before immune therapy was positive in 3 subsequent samples at 1:80, 1:160 & 1:320 pmole /l (Normal <10) immunoglobulin G (IgG) by Immunofluorescent test IFT and not detectable in the CSF. The level of VGPC autoantibodies at presentation was high in the serum at 456 pmole /l (Normal <85). The serum Contactin Associated Protein 2 (CASPR 2) was not detectable. Other autoantibodies screen was negative. She was admitted to the Epilepsy monitoring unit for further evaluation of seizure control as the seizures were poorly controlled on 4 antiepileptic drugs despite optimal doses including Carbamazepine controlled release CR at 400 milligram 2 per day, Levetiracetam one and half gram 2 per day, Lacosamide at 200 milligrams 2 per day and Phenobarbitone at 200 mg once per day. She was monitored on the same doses of the 4 antiepileptic Drugs for 5 days. A total of 24 brief stereotyped electroclinical seizures were recorded. The clinical seizures were in the form of brief left facio-brachial tonics seizures lasting for less than 10 seconds. The interictal EEG showed normal background of 8-9 hertz bilaterally intermixed with slow transients of 6-7 hertz in the temporal regions bilaterally and no epileptiform discharges seen (**Figure 1a**). The ictal EEG showed periods of right hemispheric electro decremental response with right hemispheric alpha frequency attenuation at FP2, F4, T2, T4, T6, P4 & O2 with some diffusion to the left for 4-10 seconds time locked with the left facio- brachial tonic seizures followed by recovery of the EEG background intermixed with right temporal slow transients of 4-5 hertz at T2, T4 and T6 (**Figure 1b**). Magnetic Resonance Imaging (MRI) Brain at presentation showed abnormal high signal intensity and swelling of the right hippocampus and amygdale with blurring of the margins of right amygdala and medial right temporal lobe cortex on T2 spin echo (T2SE) and fluid attenuated inversion recovery (FLAIR) images with increased diffusivity demonstrated on apparent diffusion coefficient ADC map (**Figure 2 a-c**). No abnormal enhancement on post contrast images. Positron Emission Tomogram (PET) scan of the brain showed hypermetabolic right mesial temporal area which matched with the MRI abnormality (**Figure 3**).

**Figure 4:**
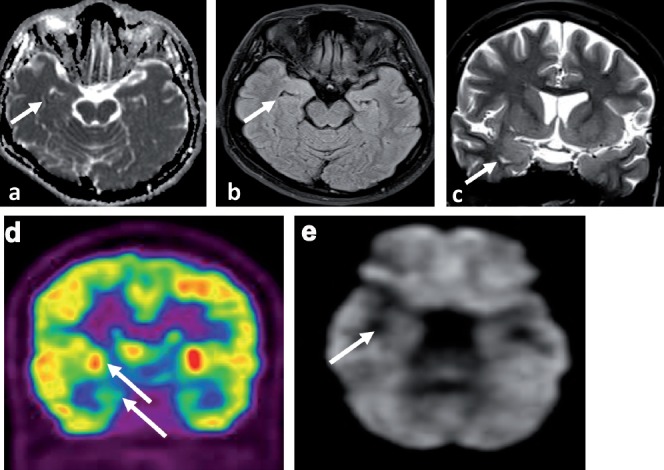
Neuroimaging at 7 months of treatment **a)** MRI brain axial ADC map, **b)** MRI brain axial FLAIR and **c)** MRI brain coronal T2SE showing almost complete resolution of the signal abnormality with no atrophic changes (white arrows). **d-e)** The PET scan of the brain showed interval resolution of the focal hypermetabolic activity previously seen in the right temporal lobe with ipsilateral relative mesial temporal and right basal ganglia hypometabolism (white arrows).

The CT scan chest, abdomen & pelvis CTCAP was negative for malignancy. Upon arrival of the LGI1 results, she was started on intravenous Methyl Prednisolone at 1 gram per day for 5 days followed by 60 milligram of oral prednisolone (1 milligram per kilogram per day) for 2 weeks then tapered gradually at 5 milligram less per week. She also received a course of Intravenous immunoglobulins (IVIG) at 0.4 gram per kilogram per day for 5 days with the oral prednisolone. This was followed by cyclic IVIG courses at 0.4 gram per kilogram per day for 5 days per month for 6 months. She showed significant improvement in seizure control and cognitive function. The antiepileptic drugs were tapered gradually. In the latest follow up, she was seizure free for 6 month on Carbamazepine (CR) 400 milligram 2 per day, Levetiracetam one gram 2 per day and lacosamide 100 milligram 2 per day to be tapered further at a later stage of follow up. Follow up EEG at 6 months was normal. Magnetic Resonance Imaging brain at 7 months after presentation showed almost complete resolution of the right temporal signal abnormalities with no atrophic changes (**Figure 4 a-c**) and PET scan of the brain showed interval resolution of the focal hypermetabolic activity previously seen in the right temporal lobe with ipsilateral relative mesial temporal and right basal ganglia hypometabolism (**Figure 4 d&e**). Leucine-rich glioma-inactivated protein 1 auto antibodies titer in the serum at 7 months significantly dropped but still positive at a titer at 1:80 pmole/l IgG - IFT, and the level of serum VGPC autoantibodies also dropped but still positive at 153 pmole/l. She is still under regular follow up in the epilepsy clinic on prednisolone 10 mg once per day and 2 antiepileptic drugs including Carbamazepine (CR) 400 mg 2 daily and Levetiracetam one gram 2 per day. Both the prednisolone and the antiepileptic drugs will be tapered slowly during the clinic follow up.

## Discussion

Autoimmune limbic encephalitis and a variety of other neurological disorders are associated with antibodies against the voltage-gated potassium channels VGKC.^1^,^2^ The VGKC is a multi-protein complex channel that has several protein antigens against which different auto antibodies are formed and accordingly different clinical syndromes appear.^3^ The proteins LGI1 and contactin-associated protein-like 2 (Caspr2) are the main antigens of the VGKC.^3^ Leucine-rich glioma-inactivated protein 1 is a neuronal secreted protein that modulates the synaptic excitability, and the Caspr2 is a neuronal surface protein that exists in different regions of the brain and myelinated axons.^4^ The LGI1 autoantibodies are more commonly associated with limbic encephalitis.^4^ The Caspr2 autoantibodies are associated with a variety of clinical syndromes including peripheral nerve hyperexcitability also known as neuromyotonia or Isaacs syndrome and a combination of peripheral nerve hyperexcitability and encephalitis also known as Morvan Syndrome.^5^ Other auto antibodies against VGKC-related proteins different from LGI1 or Caspr2 also reported.^3^,^4^-^5^ Mutations in LGI1 and Caspr2 genes are reported to result in disorders different from those induced by the auto antibodies.^6^,^7^ Leucine-rich glioma-inactivated protein 1 gene mutation is associated with an inherited type of human epilepsy known as autosomal dominant partial epilepsy with auditory features and mutations in Caspr2 gene have been reported with motor impairment, pharmaco resistant epilepsy, intellectual disability and psychiatric disorders.^6^,^7^

Leucine-rich glioma-inactivated protein 1 auto antibodies encephalitis is the most common syndrome associated with VGKC antibodies. Common manifestations are facio-brachial dystonic seizures, myoclonus, behavior changes, memory loss, confusion and disorientation, hallucinations, extrapyramidal dysfunction, dysautonomia, neuromyotonia, hyponatremia, and peripheral nerve dysfunction but critical illness and respiratory failure are less common in LGI1 auto antibodies encephalitis.^8^ Its typical clinical presentation is one of subacute limbic encephalitis with the typical manifestations appearing in any order.^3^ Epileptic seizures may be generalized, mesial temporal lobe seizures, and tonic seizures.^3^ The over presentation of tonic seizures in this condition led to consider them a highly suggestive feature in favor of LGI1 auto antibodies encephalitis in the differential diagnosis of limbic encephalitis.^9^ Classically they present with sudden brief asymmetrical predominantly tonic or dystonic contraction in the orofacial region and upper limbs.^3^ Ictal electroencephalography EEG shows correlation between these seizures and decreased alpha frequency, which is characteristic but not pathognomonic of tonic seizures.^3^ Additional studies include hyponatraemia in up to 60% of patients and was not seen in our patient.^9^ Routine CSF tests are usually normal, although some lymphocytosis and raised protein levels are seen.^9^ In MRI T2-weighted images, like our patient, 84% of the patients display a unilateral or bilateral increase in signal intensity in the medial temporal region.^9^ Associated tumours are rare, and mostly thymomas.^3^ Response to immunotherapy with steroids, intravenous immunoglobulins, or plasma exchange is seen in 70-80% of patients therefore it is important to consider LGI1 auto antibodies encephalitis early in the differential diagnosis of such cases.^8^ Recurrence of encephalitis is rare.^3^ Our case illustrates the significance of early screening for LGI1 auto antibodies encephalitis in suspected patients. Early diagnosis as illustrated in this case report leads to proper treatment with immunotherapy such as steroids and or IVIG with rapid resolution of symptoms in those patients.^9^,10

## References

[1] Graus F, Saiz A, Dalmau J (2010). Antibodies and neuronal autoimmune disorders of the CNS. J Neurol.

[2] Lancaster E, Martinez-Hernandez E, Dalmau J (2011). Encephalitis and antibodies to synaptic and neuronal cell surface proteins. Neurology.

[3] Montojo MT, Petit-Pedrol M, Graus F, Dalmau J (2015). Clinical spectrum and diagnostic value of antibodies against the potassium channel related protein complex. Neurologia.

[4] Lai M, Huijbers MG, Lancaster E, Graus F, Bataller L, Balice-Gordon R (2010). InInvestigation of LGI1 as the antigen in limbic encephalitis previously attributed to potassium channels: a case series. Lancet Neurol.

[5] Lancaster E, Huijbers MG, Bar V, Boronat A, Wong A, Martinez-Hernandez E (2011). Investigations of caspr2, an autoantigen of encephalitis and neuromyotonia. Ann Neurol.

[6] Fukata Y, Lovero KL, Iwanaga T, Watanabe A, Yokoi N, Tabuchi K (2010). Disruption of LGI1-linked synaptic complex causes abnormal synaptic transmission and epilepsy. Proc Natl Acad Sci U S A.

[7] Gregor A, Albrecht B, Bader I, Bijlsma EK, Ekici AB, Engels H (2011). Expanding the clinical spectrum associated with defects in CNTNAP2 and NRXN1. BMC Med Genet.

[8] Vincent A, Buckley C, Schott JM, Baker I, Dewar BK, Detert N (2004). Potassium channel antibody-associated encephalopathy: a potentially immunotherapy-responsive form of limbic encephalitis. Brain.

[9] Irani SR, Stagg CJ, Schott JM, Rosenthal CR, Schneider SA, Pettingill P (2013). Faciobrachial dystonic seizures: the influence of immunotherapy on seizure control and prevention of cognitive impairment in a broadening phenotype. Brain.

